# Knowledge, attitude and practice regarding metabolic complication management after solid organ transplantation among medical staff in China

**DOI:** 10.3389/fpubh.2026.1784412

**Published:** 2026-07-14

**Authors:** Peishan Cai, Meixia Huang, Zhou Shu, Xuefeng Cai, Qiuxiang Xia, Xianpeng Zeng, Fang Zeng

**Affiliations:** 1 Department of Pharmacy, Union Hospital, Tongji Medical College, Huazhong University of Science and Technology, Wuhan, China; 2 Hubei Province Clinical Research Center for Precision Medicine for Critical Illness, Wuhan, China; 3 Department of Urology Surgery, Union Hospital, Tongji Medical College, Huazhong University of Science and Technology, Wuhan, China

**Keywords:** clinical pharmacist, clinician, knowledge, attitude, practice, metabolic complications, solid organ transplantation

## Abstract

**Background:**

Metabolic complications are a major challenge after solid organ transplantation (SOT), impacting graft and patient survival. Understanding staff’s knowledge, attitude, and practice (KAP) is crucial to optimize management.

**Methods:**

Utilizing data from a nationwide convenience sample (February–April 2025) of SOT clinicians and clinical pharmacists on the post-transplant metabolic complications management, this study analyzed 43-item KAP responses from 277 participants. Analyses included descriptive statistics, Spearman’s correlation, and multinomial logistic regression to assess KAP levels and identify influencing factors.

**Results:**

Among 277 participants, the high-level rate for overall KAP was 67.5%, with high-level rates of 62.8, 88.1, and 56.3% for the knowledge, attitude, and practice dimensions, respectively. All three KAP dimensions were significantly positively correlated (r = 0.372–0.462, all *p* < 0.05). Multinomial logistic regression identified variables associated with high level of KAP. Significant positive factors included: female gender, a master’s degree, and 10–19 years of work time for high-level knowledge; both 10–19 and ≥20 years of work time for high-level attitude; being a clinical physician for high-level practice; and age 36–44 years, a master’s degree and vice senior or chief senior titles for a high-level overall KAP score. Age 45–60 years was negatively associated with high-level attitude.

**Conclusion:**

Chinese SOT care providers demonstrate positive attitudes but suboptimal knowledge and practice in post-transplant metabolic complications management. Our study identifies self-reported educational needs and perceived practice gaps that may inform future training or quality-improvement initiatives.

## 1 Introduction

Solid organ transplantation (SOT) stands as the definitive, life-saving therapeutic intervention for patients suffering from end-stage organ failure. While surgical techniques and immunosuppressive regimens have advanced remarkably, the post-transplantation period is fraught with challenges, among which metabolic complications figure prominently (1–3). The incidence of conditions such as post-transplantation diabetes mellitus (PTDM), dyslipidemia, hypertension, and metabolic syndrome is substantially higher in the transplant population compared to the general public (3–6). This elevated risk profile is largely attributable to the long-term use of immunosuppressive medications (7). These metabolic derangements are key determinants of long-term patient survival and allograft function. Their primary mechanism of detriment is the acceleration of cardiovascular disease, which remains a leading cause of mortality in long-term transplant survivors (1, 2). Consequently, the proactive, effective, and sustained management of these metabolic complications is an integral component of comprehensive post-transplant care (8–11).

Medical staff play a crucial role in the screening, diagnosis, prevention, treatment, and long-term management of metabolic complications following SOT. However, the systematic management of associated metabolic issues (7, 12) has not received commensurate attention in clinical practice and research agendas. A thorough understanding of the current knowledge, attitude, and practice (KAP) baseline, including its strengths and deficiencies, is indispensable for the development of standardized management protocols, the design of effective targeted training programs, and the strategic allocation of clinical resources.

Currently, there is a lack of validated questionnaires specifically assessing the KAP of SOT medical staff regarding post-transplant metabolic management. To address this gap, the research team developed and validated a 43-item questionnaire. Rigorous psychometric evaluation demonstrated that the questionnaire possesses satisfactory reliability and validity, making it a useful measurement tool for assessing KAP among SOT medical staff. Using this tool, this study aimed to assess the KAP levels and identify influencing factors among SOT clinicians and clinical pharmacists in China, thereby informing the development of targeted training and quality improvement strategies. This is the first KAP survey using a nationwide convenience sample on this topic in China, and to our knowledge no similar studies have been reported internationally.

## 2 Methods

### 2.1 Study design and data source

This study utilized data from a cross-sectional survey originally designed to develop and validate a KAP questionnaire. The survey was administered to SOT clinicians and clinical pharmacists across China from February to April 2025. A prior analysis focused on the psychometric validation of the instrument, confirming it as a reliable and valid questionnaire, the present analysis utilizes the finalized 43-item KAP data from the same cohort (*N* = 277) to address distinct research aims: assessing the current KAP levels and identifying the key factors influencing KAP through multinomial logistic regression. The detailed questionnaire development and validation process is provided in the Supplementary material. The final 43-item questionnaire is also provided in the Supplementary Table S1. This study was approved by the Medical Ethics Committee of Union Hospital, Tongji Medical College, Huazhong University of Science and Technology (Wuhan, China) [(2024) 0517]. All procedures followed the ethical standards of the Helsinki Declaration. Informed consent was also obtained from all participants before enrollment. This cross-sectional study was designed and reported following the principles outlined in the STROBE checklist for cross-sectional studies (Supplementary Table S2).

### 2.2 Participants and data collection

The minimum required sample size was estimated using the prevalence-based method recommended by Bujang for cross-sectional studies (13). The equation used was $n={\left(\frac{Z_{1-\alpha /2}}{d}\right)}^{2}\times p\times (1-p)$ . Assuming a high-level rate of 50%, a 95% confidence level (*Ζ* = 1.96), and a precision of 0.07, the required sample size was approximately 196 participants. To account for a potential 20% non-response rate, the target sample size was set at 235 (14). The final sample size was 277 participants. Convenience sampling was used to recruit clinicians and clinical pharmacists involved in SOT from tertiary hospitals nationwide, aiming for broad geographic coverage. Participants were required to have at least 1 year of clinical work time and provided informed consent voluntarily. Those in advanced training, rotations, or residency programs were excluded.

The data collection form comprised two sections. The first collected general information: gender, age, education, work time, profession, and technical title. The second contained the KAP questionnaire on post-transplant metabolic complications. The knowledge dimension included 19 items (0–48 scores). For single-choice questions, a correct answer received 1 point, incorrect or “not sure” score 0. For multiple-choice questions, each correct selection earned 1 point, each incorrect selection deducted 1 point, and selecting “not sure” resulted in 0 point, with the total not below 0 (15). The attitude dimension (12–60 scores) had 12 items using a five-point Likert scale, from “completely agree” scoring 5 points to “completely disagree” scoring 1 point. The practice dimension had 12 items (12–60 scores) using a five-point Likert scale, from “always” scoring 5 points to “never” scoring 1 point.

### 2.3 Data collection progress

We recruited participants from tertiary hospitals nationwide through the China Transplant Pharmacist Alliance. The survey was conducted online using the Questionnaire Network. Participants were informed about the study’s objectives, content, completion methods and guidelines. They subsequently completed the survey by scanning the QR code through WeChat. To ensure data quality and completeness, several quality control measures were implemented, including restricting submissions to one per IP address and requiring responses to all questions. The survey remained open for 3 weeks, after which access was closed. The response data were then exported to an Excel spreadsheet from the Questionnaire Network.

### 2.4 Data analysis

First, the data distribution was analyzed before performing other statistical analyses. Since the data did not follow a normal distribution according to the Kolmogorov–Smirnov test, median and interquartile range were used to describe central tendency and dispersion.

For analytical clarity and interpretation, performance benchmarks were defined *a priori* based on percentage thresholds of the maximum possible score. The theoretical total KAP score, aggregating all dimensions, spanned from 24 to 168 points. We classified participants into three levels based on their scores: low-level for scores<60% of the maximum score, medium-level for scores between 60 and 80%, and high-level for scores≥80% (16). These cut-offs are operational benchmarks based on the commonly used grading system in Chinese medical education and are not formally validated competency standards.

### 2.5 Statistical analysis

All collected data were downloaded from the survey platform and analyzed using Microsoft Excel 2019 for initial management and IBM SPSS Statistics (version 27.0) for statistical analyses. Categorical variables were summarized as frequencies and percentages. For continuous variables, normally distributed data were expressed as mean ± standard deviation (SD). While non-normally distributed continuous data were expressed as the median and interquartile range. Independent samples t-tests and one-way analysis of variance (ANOVA) were performed for univariate comparisons of normally distributed data, while the Mann–Whitney U test and Kruskal-Wallis H test were applied for non-parametric data.

Spearman’s correlation analysis was conducted to assess the associations among knowledge, attitude and practice scores. Variables with a *p*-value of less than 0.2 in the univariate analysis were included in the multinomial logistic analysis. Multinomial logistic regression was employed to identify factors associated with different performance levels (low, medium, high) for the overall KAP score and each KAP dimension score separately. For all models, the low and medium levels were, respectively, designated as the reference categories. Model parameters were estimated using the maximum likelihood method. The threshold for statistical significance in the final multivariate models was set at *p* < 0.05. In the attitude model, quasi-complete separation occurred for technical title. We therefore merged the primary and moderate title categories into the reference category (17, 18). Multicollinearity among the independent variables was assessed using the Variance Inflation Factor (VIF). A VIF > 5 was considered indicative of problematic collinearity.

## 3 Results

### 3.1 Demographic characteristics of participants

A total of 305 questionnaires were distributed. After excluding 28 invalid questionnaires from participants who were not SOT medical staff, 277 valid questionnaires were included in the final analysis, yielding an effective response rate of 90.82%. Participants came from 27 provinces across China (including Hubei, Guangdong, Beijing, Shanghai, Zhejiang, Jiangsu, etc.), covering six types of solid organ transplantation: kidney, liver, heart, lung, pancreas, and small intestine transplantation. The cohort was 67.5% male, with the largest age group being 25–35 years (45.5%). Most participants held a master’s degree (55.2%) and held a moderate professional title (52.7%). Nearly half (49.1%) had 1–9 years of clinical experience, and clinicians constituted the majority (77.6%) of the sample. Detailed demographic characteristics are presented in Table 1.

**Table 1:** Univariate analysis of KAP score by demographic factors.

Item	*N* (%)	Knowledge dimension M (P25, P75)	Statistic (z/*χ*^2^)	*p*	Attitude dimension M (P25, P75)	Statistic (z/*χ*^2^)	*p*	Practice dimension M (P25, P75)	Statistic (z/*χ*^2^)	*p*	Total score M (P25, P75)	Statistic (z/*χ*^2^)	*p*
Gender			3.291	0.001		1.342	0.180		−1.390	0.164		0.984	0.325
Male	187 (67.5)	40.00 (34.00,45.00)			58.00 (48.00,60.00)			48.00 (43.00,56.00)			144.00 (128.00,156.50)		
Female	90 (32.5)	43.00 (39.00,46.00)			60.00 (48.00,60.00)			47.00 (41.00,55.00)			144.50 (132.00,156.00)		
Age (years)			1.694	0.429		4.437	0.109		6.264	0.044		5.851	0.054
25–35	126 (45.5)	41.00 (33.00,45.00)			58.00 (48.00,60.00)			47.00 (40.00,55.00)			140.00 (125.00,153.00)		
36–44	105 (37.9)	41.00 (35.00,45.00)			59.00 (50.00,60.00)			50.00 (44.00,55.00)			146.00 (132.00,156.00)		
45–60	46 (16.6)	42.50 (36.00,46.00)			57.00 (48.00,60.00)			51.50 (47.00,57.00)			149.50 (135.00,158.00)		
Education			9.150	0.010		2.328	0.312		0.997	0.608		4.621	0.099
Bachelor’s degree	39 (14.1)	38.00 (30.00,45.00)			56.00 (48.00,60.00)			48.00 (37.50,55.50)			140.00 (120.50,150.00)		
Master’s degree	153 (55.2)	42.00 (37.00,45.00)			59.00 (49.00,60.00)			48.00 (43.00,55.00)			145.00 (132.00,157.00)		
Doctor’s degree	85 (30.7)	40.00 (32.00,44.00)			58.00 (48.00,60.00)			49.00 (43.00,56.00)			146.00 (128.00,155.00)		
Technical Title			3.273	0.351		5.096	0.165		6.861	0.076		9.412	0.024
Primary	29 (10.5)	42.00 (37.00,44.00)			50.00 (48.00,60.00)			46.00 (41.00,53.00)			139.00 (127.00,152.00)		
Moderate	146 (52.7)	41.00 (33.00,45.00)			58.00 (48.00,60.00)			48.00 (42.00,55.00)			141.00 (129.00,153.00)		
Vice senior	80 (28.9)	42.00 (37.00,45.00)			60.00 (49.50,60.00)			51.00 (44.50,57.50)			149.50 (141.00,158.00)		
Chief senior	22 (7.9)	37.00 (34.00,45.00)			53.50 (48.00,60.00)			51.00 (42.00,57.00)			145.50 (128.00,156.00)		
Work time (years)			3.494	0.174		5.704	0.058		8.993	0.011		9.498	0.009
1–9	136 (49.1)	41.00 (33.00,45.00)			57.00 (48.00,60.00)			47.00 (41.00,55.00)			140.00 (126.50,153.50)		
10–19	106 (38.3)	42.00 (36.00,45.00)			60.00 (49.00,60.00)			49.00 (42.00,55.00)			146.50 (135.00,156.00)		
≥20	35 (12.6)	43.00 (36.50,46.00)			60.00 (48.00,60.00)			52.00 (47.50,59.00)			152.00 (144.50,159.00)		
Profession			2.415	0.016		1.701	0.089		−2.685	0.007		−0.025	0.980
Clinician	215 (77.6)	41.00 (34.00,45.00)			58.00 (48.00,60.00)			48.00 (43.00,56.00)			145.00 (129.00,157.00)		
Clinical pharmacist	62 (22.4)	43.00 (38.00,46.00)			60.00 (51.00,60.00)			46.00 (39.00,53.00)			143.00 (132.00,155.00)		

### 3.2 Overall KAP scores and distribution

The overall questionnaire, knowledge, attitude, and practice median scores were 144.00 (129.00, 156.00), 41.00 (34.50, 45.00), 59.00 (48.00, 60.00), and 48.00 (42.00, 56.00), respectively. Overall, 93.5% (259/277) of participants achieved at least a medium-level (medium or high), and 67.5% (187/277) attained a high-level score. Disaggregated analysis showed that the attitude dimension had the highest proportion of high-level participants (88.1%), followed by the medium-level (7.9%) and low-level (4.0%). In contrast, the practice dimension had a lower high-level rate (56.3%), despite a medium-level rate of 35.8%. The knowledge dimension had a high-level rate of 62.8% and a medium-level rate of 24.6% (Table 2).

**Table 2:** Knowledge, attitude, and practice scores of medical staff regarding metabolic complications management after solid organ transplantation.

Dimension	Number of entries	Score range	Minimum score	Maximum score	Score M	Low level	Medium level	High level
					(P25, P75)	rate N (%)	rate N (%)	rate N (%)
Knowledge	19	0 ~ 48	0	48	41.00 (34.50,45.00)	35(12.6)	68(24.6)	174 (62.8)
Attitude	12	12 ~ 60	12	60	59.00 (48.00,60.00)	11(4.0)	22(7.9)	244(88.1)
Practice	12	12 ~ 60	12	60	48.00 (42.00,56.00)	22(7.9)	99(35.8)	156(56.3)
Overall	43	24 ~ 168	24	168	144.00 (129.00,156.00)	18(6.5)	72 (26.0)	187 (67.5)

Variables with a *p*-value < 0.2 in the univariate analyses (Table 1) were considered candidates for inclusion in the multivariate models. These included gender, education, work time and profession for knowledge dimension; gender, age, technical title, work time and profession for attitude dimension; gender, age, technical title, work time and profession for practice dimension; and age, education, technical title, and work time for overall KAP.

### 3.3 The scores of the items in the dimensions of KAP

Item-level responses for KAP dimensions are presented in Tables 3–5. Five knowledge items had low correct rates: K9 (11.6%), K7 (36.8%), K15 (43.7%), K10 (47.3%), and K8 (49.1%). These knowledge gaps were mainly in pharmacotherapy: precise therapeutic goal setting, drug-specific adverse effect profiling, detailed therapeutic monitoring, and contraindication assessment. Furthermore, clinical physicians consistently scored lower than clinical pharmacists across all these items (K9: 7.9% vs. 24.1%; K7: 33.0% vs. 50.0%; K15: 39.0% vs. 59.6%; K10: 46.5% vs. 50.0%; K8: 42.7% vs. 70.9%). Attitude responses were uniformly positive. All items had a median score of 5.00 (4.00, 5.00), with 58.5–64.3% of participants selecting the most positive response (“completely agree”). Negative responses (“completely disagree” or “disagree”) were rare (<5% for every item). In contrast, reported practice behaviors were more modest and varied. While the median score for all items was 4.00, the interquartile range (3.00, 5.00) for several items indicated considerable response variation. The proportion of participants who “always” performed recommended practices (score of 5) ranged from 18.8 to 41.9%, markedly lower than the “completely agree” proportions for attitudes. Meanwhile, clinicians consistently had higher “always” rates than clinical pharmacists across all 12 items (Supplementary Table S3).

**Table 3:** Correct rates of items related to the knowledge dimension.

Question	Correct rate (%)
K1. The high incidence of metabolic diseases after transplantation significantly affects the recipient’s quality of life and long-term survival.	262 (94.6%)
K2. What are the risk factors associated with metabolic diseases after transplantation?	151 (54.5%)
K3. What are the common metabolic complications in transplant recipients?	227 (81.9%)
K4. Transplant recipients should routinely undergo screening for blood pressure, blood glucose, blood lipids, and uric acid levels.	262 (94.6%)
K5. The target for blood pressure control in transplant recipients should be individualized based on clinical circumstances.	261 (94.2%)
K6. ACEI or ARB medications have the effect of reducing urinary protein.	249 (89.9%)
K7. Which of the following are common adverse reactions of CCB drugs?	102 (36.8%)
K8. Which of the following parameters should be regularly monitored during the use of ACEIs and ARBs?	136 (49.1%)
K9. Regarding the long-term blood glucose control targets for PTDM patients, which of the following is correct?	32 (11.6%)
K10. In which of the following situations is metformin contraindicated?	131 (47.3%)
K11. What are the indications for initiating insulin therapy in patients with PTDM?	155 (56.0%)
K12. Dyslipidemia in transplant recipients refers to elevated levels of TC and/or TG in the serum, as well as various lipid abnormalities including LDL-C and HDL-C.	257 (92.8%)
K13. The goals and intensity of lipid-lowering therapy for post-transplant patients with dyslipidemia should be determined based on their ASCVD risk stratification.	242 (87.4%)
K14. When pharmacologic therapy is indicated for hypercholesterolemia in transplant recipients, statins are the first-line agents.	246 (88.8%)
K15. Which of the following parameters should be monitored during statin therapy?	121 (43.7%)
K16. The intervention threshold for hyperuricemia after transplantation is: serum uric acid >420 μmol/L in men and >360 μmol/L in women.	243 (87.7%)
K17. Regarding the control targets for hyperuricemia after transplantation, which of the following statements are correct?	175 (63.2%)
K18. Which of the following are intervention measures for metabolic disorders in transplant recipients?	227 (81.9%)
K19. What do lifestyle modifications for transplant recipients include?	241 (87.0%)

**Table 4:** The scores of items in attitude dimension.

Item	Scores M (P25, P75)	1 point N (%)	2 points N (%)	3 points N (%)	4 points N (%)	5 points N (%)
A1: Timely diagnosis and treatment of metabolic diseases in transplant recipients can prevent and delay the progression of metabolic complications, effectively improving long-term outcomes for recipients.	5.00 (4.00,5.00)	13 (4.7)	8 (2.9)	9 (3.2)	85 (30.7)	162 (58.5)
A2: Physicians and pharmacists should actively study guidelines or consensus statements on the prevention and management of metabolic disorders in transplant recipients in order to provide better clinical care.	5.00 (4.00,5.00)	7 (2.5)	7 (2.5)	10 (3.6)	75 (27.1)	178 (64.3)
A3: Establishing standardized procedures for managing metabolic diseases in transplant recipients is very important.	5.00 (4.00,5.00)	7 (2.5)	5 (1.8)	7 (2.5)	86 (31.0)	172 (62.1)
A4: Evaluating risk factors associated with metabolic diseases in transplant recipients and implementing active interventions can reduce their incidence risk.	5.00 (4.00,5.00)	8 (2.9)	6 (2.2)	7 (2.5)	83 (30.0)	173 (62.5)
A5: Metabolic-related indicators should be regularly monitored for every transplant recipient.	5.00 (4.00,5.00)	5 (1.8)	4 (1.4)	13 (4.7)	83 (30.0)	172 (62.1)
A6: Personalized guidance on diet and exercise should be provided to patients.	5.00 (4.00,5.00)	4 (1.4)	3 (1.1)	15 (5.4)	92 (33.2)	163 (58.8)
A7: The scientific selection and appropriate adjustment of immunosuppressants for transplant recipients are important components of metabolic disease management.	5.00 (4.00,5.00)	6 (2.2)	3 (1.1)	8 (2.9)	89 (32.1)	171 (61.7)
A8: If lifestyle modifications and adjustments to the immunosuppressive regimen still fail to bring metabolic indicators of transplant recipients to target levels, pharmacological treatment should be initiated promptly.	5.00 (4.00,5.00)	7 (2.5)	3 (1.1)	12 (4.3)	92 (33.2)	163 (58.8)
A9: Potential interactions between medications for metabolic diseases and immunosuppressants should be thoroughly understood.	5.00 (4.00,5.00)	7 (2.5)	3 (1.1)	9 (3.2)	85(30.7)	173 (62.5)
A10: Adverse reactions to medications used for treating metabolic diseases in transplant recipients should be proactively monitored.	5.00 (4.00,5.00)	7 (2.5)	2 (0.7)	5 (1.8)	93 (33.6)	170 (61.4)
A11: It is very important to regularly provide transplant recipients with education on metabolic diseases.	5.00 (4.00,5.00)	5 (1.8)	3 (1.1)	9 (3.2)	86 (31.0)	174 (62.8)
A12: In clinical practice, establish open, effective communication with recipients, listen to their needs and concerns, and offer reassurance and hope.	5.00 (4.00,5.00)	3 (1.1)	4 (1.4)	11 (4.0)	83 (30.0)	176 (63.5)

**Table 5:** The Scores of items in practice dimension.

Item	Scores M (P25, P75)	1 point N (%)	2 points N (%)	3 points N (%)	4 points N (%)	5 points N (%)
P1: Do you proactively learn knowledge about the prevention and treatment of metabolic diseases in transplant recipients?	4.00 (3.00,4.00)	2 (0.7)	16 (5.8)	78 (28.2)	129 (46.6)	52 (18.8)
P2: Do you regularly monitor the blood pressure, blood glucose, lipid levels, and uric acid levels of transplant recipients?	4.00 (4.00,5.00)	3 (1.1)	16 (5.8)	36 (13.0)	106 (38.3)	116 (41.9)
P3: Will you assess the risk of metabolic diseases in transplant recipients and take appropriate measures?	4.00 (4.00,5.00)	2 (0.7)	18 (6.5)	46 (16.6)	117 (42.2)	94 (33.9)
P4: Do you actively intervene in transplant patients with abnormal metabolic indicators?	4.00 (4.00,5.00)	3 (1.1)	11 (4.0)	47 (17.0)	116 (41.9)	100 (36.1)
P5: Will you evaluate the treatment efficacy for metabolic diseases in transplant recipients?	4.00 (4.00,5.00)	3 (1.1)	12 (4.3)	46 (16.6)	113 (40.8)	103 (37.2)
P6: Do you develop individualized control goals for transplant patients with metabolic diseases?	4.00 (3.00,5.00)	2 (0.7)	19 (6.9)	56 (20.2)	108 (39.0)	92 (33.2)
P7: Do you provide dietary and exercise guidance for patients with post-transplant metabolic diseases?	4.00 (3.00,5.00)	3 (1.1)	14 (5.1)	62 (22.4)	103 (37.2)	95 (34.3)
P8: Do you consider adjusting immunosuppressants as a key strategy in managing post-transplant metabolic diseases?	4.00 (3.00,5.00)	5 (1.8)	16 (5.8)	82 (29.6)	92 (33.2)	82 (29.6)
P9: Do you assess the interactions between medications for metabolic diseases and immunosuppressants in transplant recipients?	4.00 (4.00,5.00)	2 (0.7)	7 (2.5)	56 (20.2)	112 (40.4)	100 (36.1)
P10: Do you monitor for adverse reactions to medications used to treat metabolic diseases in transplant recipients?	4.00 (4.00,5.00)	1 (0.4)	14 (5.1)	44 (15.9)	118 (42.6)	100 (36.1)
P11: Do you provide education to transplant recipients about metabolic complications?	4.00 (3.00,5.00)	1 (0.4)	17 (6.1)	61 (22.0)	111 (40.1)	87 (31.4)
P12: During consultations, do you patiently listen to patients’feelings and concerns and provide them with emotional support?	4.00 (3.00,5.00)	1 (0.4)	14 (5.1)	56 (20.2)	107 (38.6)	99 (35.7)

### 3.4 Correlations among KAP dimensions

Spearman’s correlation analysis (Table 6) revealed that the knowledge score showed positive correlations with the attitude score (*r* = 0.390, *p* < 0.001) and practice score (*r* = 0.372, *p* < 0.001). There was also a positive correlation between the attitude and practice scores (*r* = 0.462, *p* < 0.001).

**Table 6:** Spearman correlation coefficients for knowledge, attitude, and practice scores.

Dimension	Knowledge	Attitude	Practice
Knowledge	1.000		
Attitude	0.390 (*p* < 0.001)	1.000	
Practice	0.372 (*p* < 0.001)	0.462 (*p* < 0.001)	1.000

### 3.5 Analysis of factors influencing KAP

Collinearity diagnostics showed that all VIF values were below 5 (range 1.141–3.365), indicating no severe multicollinearity among the variables (Table 7). Multinomial logistic regression analyses, with low-level as the reference category and adjusted for confounders, identified variables associated with medium-level and high-level outcomes. In the medium-level and low-level comparison (Table 8), higher odds of medium-level were associated with: 10–19 years of work time for knowledge (OR = 2.716, 95% CI 1.015–7.272, *p* = 0.047); female gender (OR = 6.554, 95% CI 1.129–38.057, *p* = 0.036) and work time≥20 years (OR = 24.096, 95% CI 1.266–458.451, *p* = 0.034) for attitude, whereas age 45–60 years showed lower odds (OR = 0.072, 95% CI 0.006–0.826, *p* = 0.035). No factors were significantly associated with medium-level practice. For the overall KAP score, participants aged 36–44 years had higher odds of medium-level compared to the reference age group (OR = 29.976, 95% CI 2.544–353.192, *p* = 0.007).

**Table 7:** Collinearity diagnostics (VIF) for independent variables.

Variable	Tolerance	VIF
Gender	0.677	1.477
Age	0.297	3.365
Education	0.876	1.141
Work time	0.315	3.173
Technical title	0.505	1.98
Profession	0.667	1.499

**Table 8:** Multinomial logistic regression analysis (medium-level vs. low-level) of factors influencing KAP.

Factors	Knowledge		Attitude		Practice		Overall	
	OR (95% CI)	*p*	OR (95% CI)	*p*	OR (95% CI)	*p*	OR (95% CI)	*p*
Gender								
Female	1.952 (0.576,6.619)	0.283	6.554 (1.129,38.057)	0.036	1.137 (0.496,2.609)	0.761		
Male	Ref.		Ref.		Ref.			
Age (years)								
45–60			0.072 (0.006,0.826)	0.035	1.006 (0.181,5.602)	0.994	2.075 (0.170,25.272)	0.567
36–44			1.666 (0.250,11.107)	0.598	1.454 (0.519,4.071)	0.476	29.976 (2.544,353.192)	0.007
25–35			Ref.		Ref.		Ref.	
Education								
Doctor’s degree	1.518 (0.463,4.980)	0.491					1.432 (0.378,5.417)	0.597
Master’s degree	1.943 (0.605,6.243)	0.265					2.637 (0.690,10.075)	0.156
Bachelor’s degree	Ref.						Ref.	
Work time (years)								
≥20	1.194 (0.334,4.273)	0.785	24.096 (1.266,458.451)	0.034	1.342 (0.185,9.747)	0.771	2.996 (0.238.37.731)	0.396
10–19	2.716 (1.015,7.272)	0.047	9.596 (0.970,94.908)	0.053	0.811 (0.288,2.287)	0.693	1.914 (0.345,10.612)	0.457
1–9	Ref.		Ref.		Ref.		Ref.	
Technical title								
Chief senior			2.208 (0.463,10.528)	0.320	0.903 (0.132,6.193)	0.918	0.063 (0.004,1.123)	0.060
Vice senior			0.243 (0.048,1.218)	0.085	1.730 (0.415,7.211)	0.452	0.121 (0.012,1.181)	0.069
Moderate			Ref.^a^		0.962 (0.331,2.793)	0.943	0.407 (0.074,2.237)	0.301
Primary					Ref.		Ref.	
Profession								
Clinician	1.794 (0.494,6.513)	0.374	1.613 (0.241,10.799)	0.622	2.196 (0.900,5.357)	0.084		
Clinical pharmacist	Ref.		Ref.		Ref.			

^a^Due to zero frequency in the attitude “low-level” category (quasi-complete separation), the primary technical title was combined with the moderate title as the reference group.

Meanwhile, in the high-level and low-level comparison (Table 9), associations with high-level differed across dimensions. For knowledge, a high level of knowledge was significantly associated with female gender (OR = 4.725, 95% CI 1.544–14.465, *p* = 0.007), holding a master’s degree (OR = 5.842, 95% CI 1.942–17.579, *p* = 0.002), and 10–19 years of work time (OR = 3.004, 95% CI 1.194–7.557, *p* = 0.019). For attitude, high-level was associated with both 10–19 years (OR = 12.196, 95% CI 1.307–113.806, *p* = 0.028) and ≥20 years of work time (OR = 48.537, 95% CI 2.886–816.411, *p* = 0.007), whereas age 45–60 years was associated with lower odds (OR = 0.046, 95% CI 0.004–0.484, *p* = 0.010). For practice, being a clinical physician was associated with higher odds of high-level (OR = 2.897, 95% CI 1.117–7.516, *p* = 0.029). Finally, a high-level overall KAP score was positively associated with the 36–44 years age group (OR = 23.528, 95% CI 2.087–265.192, *p* = 0.011), possession of a master’s degree (OR = 6.610, 95% CI 1.812–24.106, *p* = 0.004), whereas holding a chief senior technical title (OR = 0.024, 95% CI 0.002–0.397, *p* = 0.009) was associated with lower odds. However, this estimate is unstable due to sparse data (only three chief senior participants in the low-level category) and should not be interpreted as a reliable negative association.

**Table 9:** Multinomial logistic regression analysis (high-level vs. low-level) of factors influencing KAP.

Factors	Knowledge		Attitude		Practice		Overall	
	OR (95% CI)	*p*	OR (95% CI)	*p*	OR (95% CI)	*p*	OR (95% CI)	*p*
Gender								
Female	4.725 (1.544,14.465)	0.007	5.110 (0.918,28.437)	0.063	0.937 (0.394,2.226)	0.882		
Male	Ref.		Ref.		Ref.			
Age (years)								
45–60			0.046 (0.004,0.484)	0.010	0.585 (0.095,3.605)	0.564	3.325 (0.324,34.165)	0.312
36–44			1.614 (0.261,9.991)	0.607	1.736 (0.598,5.034)	0.310	23.528 (2.087,265.192)	0.011
25–35			Ref.		Ref.		Ref.	
Education								
Doctor’s degree	2.453 (0.791,7.605)	0.120					1.783 (0.494,6.437)	0.377
Master’s degree	5.842 (1.942,17.579)	0.002					6.610 (1.812,24.106)	0.004
Bachelor’s degree	Ref.						Ref.	
Work time (years)								
≥20	1.767 (0.564,5.532)	0.328	48.537 (2.886,816.411)	0.007	4.541 (0.609,33.866)	0.140	3.119 (0.285,34.134)	0.351
10–19	3.004 (1.194,7.557)	0.019	12.196 (1.307,113.806)	0.028	0.867 (0.294,2.556)	0.796	2.380 (0.457,12.404)	0.303
1–9	Ref.		Ref.		Ref.		Ref.	
Technical title								
Chief senior			1.017 (0.218,4.744)	0.983	1.392 (0.200,9.675)	0.738	0.024 (0.002,0.397)	0.009
Vice senior			0.363 (0.079,1.668)	0.193	2.615 (0.593,11.525)	0.204	0.175 (0.020,1.541)	0.116
Moderate			Ref.^b^		1.080 (0.344,3.391)	0.895	0.370 (0.072,1.914)	0.236
Primary					Ref.		Ref.	
Profession								
Clinician	1.612 (0.508,5.117)	0.418	0.847 (0.137,5.234)	0.858	2.897 (1.117,7.516)	0.029		
Clinical pharmacist	Ref.		Ref.		Ref.			

^a^Due to zero frequency in the attitude “low-level” category (quasi-complete separation), the primary technical title was combined with the moderate title as the reference group.

For the overall KAP score, compared with the low-level, the high-level was positively associated with vice senior (OR = 4.982, 95% CI 1.627–15.255, *p* = 0.005) and chief senior (OR = 5.646, 95% CI 1.321–24.126, *p* = 0.020). These estimates may be more informative but should be interpreted cautiously. No significant associations were observed for the knowledge, attitude, or practice dimensions (Table 10).

**Table 10:** Multinomial logistic regression analysis (high-level vs. medium-level) of factors influencing KAP.

Factors	Knowledge		Attitude		Practice		Overall	
	OR (95% CI)	*p*	OR (95% CI)	*p*	OR (95% CI)	*p*	OR (95% CI)	*p*
Gender								
Female	2.286 (0.972,5.377)	0.058	0.769 (0.379,1.561)	0.467	0.545 (0.800,0.389)	0.545		
Male	Ref.		Ref.		Ref.			
Age (years)								
45–60			0.529 (0.115,2.438)	0.414	0.840 (0.195,3.612)	0.815	1.330 (0.259,6.827)	0.733
36–44			1.136 (0.451,2.861)	0.786	1.359 (0.535,3.451)	0.519	0.614 (0.243,1.551)	0.302
25–35			Ref.		Ref.		Ref.	
Education								
Doctor’s degree	1.282 (0.467,3.517)	0.630					2.354 (0.898,6.173)	0.082
Master’s degree	2.318 (0.868,6.190)	0.093					2.260 (0.925,5.523)	0.074
Bachelor’s degree	Ref.						Ref.	
Work time (years)								
≥20	1.650 (0.610,4.464)	0.324	3.164 (0.555,18.020)	0.194	3.614 (0.736,17.750)	0.114	4.311 (0.608,30.564)	0.144
10–19	1.145 (0.571,2.296)	0.703	1.194 (0.437,3.561)	0.729	1.104 (0.434,2.807)	0.835	2.030 (0.786,5.245)	0.144
1–9	Ref.		Ref.		Ref.		Ref.	
Technical title								
Chief senior			0.697 (0.179,2.715)	0.602	1.497 (0.395,5.677)	0.553	5.646 (1.321,24.126)	0.020
Vice senior			1.566 (0.620,3.959)	0.343	1.294 (0.433,3.865)	0.644	4.982 (1.627,15.255)	0.005
Moderate			Ref.^b^		1.443 (0.517,4.024)	0.483	2.182 (0.072,1.914)	0.116
Primary					Ref.		Ref.	
Profession								
Clinician	0.631 (0.187,2.125)	0.457	0.577 (0.219,1.525)	0.268	1.040 (0.385,2.808)	0.938		
Clinical pharmacist	Ref.		Ref.		Ref.			

^a^Due to zero frequency in the attitude “low-level” category (quasi-complete separation), the primary technical title was combined with the moderate title as the reference group.

Notably, consistent associations were observed for specific factors across different outcome levels. Work time of 10–19 years was positively associated with both medium and high knowledge levels. Similarly, work time of ≥20 years was associated with higher odds for both medium and high attitude levels, and the 36–44 years age group was associated with both medium and high levels in the overall KAP scores. In contrast, the 45–60 years age group was consistently associated with lower odds of positive attitude outcomes.

## 4 Discussion

This first KAP survey using a nationwide convenience sample among Chinese SOT clinicians and clinical pharmacists provides baseline data on the management of post-transplant metabolic complications. It reveals a profile: despite strongly positive attitudes, a gap exists between the high-level rates for attitudes and those for knowledge and practice. The identification of distinct factors associated with excellence across KAP dimensions further clarifies the specific contours of these gaps. These findings may help inform future training and collaborative care models, with the aim of supporting high-quality practice.

The proportions of participants achieving at least a medium-level were high (all >87% across KAP dimensions and the overall score), suggesting widespread foundational awareness (10). However, the high-level rates varied considerably across dimensions: attitudes had the highest high-level rate (88.1%), followed by knowledge (62.8%), practice (56.3%) and the overall KAP score (67.5%). These results suggest that most staff have achieved basic competency, but further improvement is needed to reach a high-level, particularly in practice. The questionnaire used in this study was scientifically developed and validated to effectively measure KAP among SOT clinicians and clinical pharmacists regarding the management of post-transplant metabolic complications. Therefore, the observed variation in high-level rates across dimensions reflects perceived rather than directly observed differences in clinical performance. Nonetheless, these perceived gaps may still inform efforts to improve care quality, pending confirmation by objective indicators. To bridge this performance gap, a multifaceted strategy could be considered. First, for knowledge depth, continuing medical education could shift from awareness to mastery-focused training, including case-based workshops and simulations on complex pharmacotherapy (e.g., glycemic targets, adverse reaction management). Second, to address the practice gap, system-level interventions should lower barriers to action. For clinicians, embedding guideline essentials into electronic health records as decision support or order sets could make best practices default. For pharmacists, formalizing their role through pharmacist-led metabolic clinics or embedded pharmaceutical care pathways could leverage their expertise in areas where physicians’ knowledge is weaker. Together, these coordinated interventions might help address the observed gaps, though their impact on long-term outcomes remains to be evaluated.

The low correct rates on five specific pharmacotherapy items suggest that SOT clinicians and clinical pharmacists’ knowledge of key therapeutic details is incomplete, which may hinder their ability to make precise clinical decisions. These items are related to glycemic control targets (K9) (19, 20), specific adverse drug reactions (K7) (21), detailed therapeutic monitoring requirements (K8, K15) (22, 23), and precise medication contraindications (K10) (9, 24). Addressing these knowledge deficits is important for improving the quality and safety of medication management. Notably, clinical pharmacists had higher correct rates than physicians on these five items, consistent with their training in pharmaceutical sciences and pharmacovigilance (9, 25–28). Physicians’ lower performance reflects a role-specific gap, as their clinical focus may prioritize immediate patient stabilization over detailed pharmacotherapy knowledge. These findings suggest that pharmacists’ expertise could be valuable within transplant teams. Integrating pharmacists into routine rounds might support real-time medication optimization and patient counseling, which may help reduce adverse drug events and improve metabolic control, though these potential benefits require confirmation in longitudinal studies.

SOT clinicians and clinical pharmacists showed consistently positive attitudes across all items, aligning with the “lifelong health management” model advocated by the ESOT (29) and reflecting openness among Chinese providers. This collective buy-in is essential for successful practice change (30).

However, item-level analysis reveals a disparity between highly positive attitudes and only moderately optimal practices, indicating an “intention-behavior gap.” This gap may be discussed in light of behavioral change models such as the Transtheoretical Model (TTM), which views behavior change as progressing through stages, although this framework was not directly tested in our study (31). Many providers appear to be in the contemplation or preparation stages; they are aware of guidelines and motivated to change, but have not yet acted consistently. To facilitate this transition, interventions could lower the implementation threshold. For example, by embedding specific “if-then” prompts into clinical workflows (32). Such an approach might help translate guideline recommendations into automatic actions, potentially bridging the intention-behavior gap.

The positive correlation among knowledge, attitude, and practice scores is consistent with the basic logic of the KAP model. However, the weak-to-moderate correlations (*r* = 0.372–0.462) suggest that the translation of positive attitudes into consistent practice is not automatic. This disconnect may be discussed using the Health Belief Model (HBM), which identifies self-efficacy (confidence to execute a behavior) as a potential moderating factor, although the model was not directly tested here (33, 34). Medical staff’s self-efficacy may be reduced by lack of confidence in managing complex complications, concerns about medication side effects, and absence of timely positive feedback. Interventions that target self-efficacy, such as simulation training and case reviews, might help build competence and confidence. Sharing successful management experiences and formally recognizing positive outcomes might also provide reinforcing feedback.

Multinomial logistic regression analyses indicate that female gender, a master’s degree, and 10–19 years of work time were associated with higher knowledge performance in this exploratory sample. The gender difference may reflect communication styles. Female physicians tend to use more patient-centered communication, which could facilitate knowledge acquisition (35, 36). The association with a master’s degree (OR = 5.842) suggests that structured postgraduate training may help build specialized knowledge and critical appraisal skills (37). The positive link with 10–19 years of work experience points to professional socialization; repeated exposure to complex cases may enhance self-efficacy and deepen appreciation of metabolic management (38). These findings suggest that targeted education could be considered, especially for early-career staff and those without advanced degrees, to help close the knowledge gap.

For attitude, age 45–60 was associated with lower odds, whereas longer work time (10–19 and ≥20 years) was positively associated. Female gender was also positively associated with medium-level attitude (OR = 6.554), consistent with literature on gender differences in communication (35, 36). Work experience suggested an exploratory association: staff with 10–19 and ≥20 years had higher odds of high-level attitude (OR = 12.20 and 48.54, respectively). This pattern suggests that sustained clinical exposure may help internalize proactive management values into professional identity (39, 40). To optimize team-based metabolic management, forming interdisciplinary teams that mix diverse experience levels could be beneficial, combining the expertise of senior staff with the updated knowledge of junior colleagues. The lower likelihood of high or medium level attitudes in the 45–60 age group warrants further investigation. Although long work time appeared to be associated with higher odds, age 45–60 appeared to be associated with lower odds. Age and work time are not fully collinear (VIF < 5), and 30.5% of participants aged 45–60 had <20 years of work time, indicating they capture different aspects of seniority. Burnout, which is reportedly higher in this career stage (41, 42), might contribute, along with evolving professional roles or differences in adapting to newer management paradigms. For this senior group, diagnostic evaluations could help identify causes of burnout and inform tailored support to mitigate attrition and preserve experience capital.

In behavioral outcomes, clinical physician status was associated with higher odds of high-level practice (OR = 2.897). Their authority to prescribe and adjust treatment may give them a greater sense of control over clinical actions, which can be discussed in the context of the Theory of Planned Behavior (TPB), though this framework was not directly tested in our study (43, 44). Item-level analysis confirmed clinicians outperformed pharmacists on all practice items. Pharmacists, despite deeper drug knowledge, lack such authority due to undefined roles and limited collaboration (45–47). To address this practice gap, we suggest a phased pathway based on domestic precedents. In the short term, transplant centers could adopt the Guangdong pharmacy clinic model, which has been incorporated into national service standards and supports the development of transplant medication management clinics (48). In the medium term, hospital-level Collaborative Drug Therapy Management (CDTM) agreements could be formalized, supported by a Chinese RCT showing improved medication compliance and quality of life (49). Long-term advocacy for pharmacist prescribing authority could leverage Guangdong’s “Chief Pharmacist System” pilot (50). Although barriers remain (51), the growth of CDTM in U. S. hospitals (from 50 to 66%) and emerging policy support in China suggest that a stepwise approach may be feasible (52).

A high-level overall KAP score was associated with age 36–44 years, a master’s degree, and senior titles. The 36–44 age group appeared to be associated with both moderate and high total scores, which may be interpreted in light of Benner’s mid-career progression from competence to proficiency (53, 54), and peak cognitive flexibility (55). The association with a master’s degree suggests the value of advanced training. Continuing medical education could shift toward case-based and simulation-driven programs. As noted in the Results, the negative association of chief senior titles in the high vs. low level was unstable due to sparse data. In the high vs. medium level analysis, both vice senior and chief senior were positive associations, suggesting that among those who reached at least medium-level, senior professionals were more likely to achieve high-level. Rather than viewing senior experts as resistant to updates, we could pair them with younger colleagues proficient in new guidelines and offer focused workshops to support their learning (56, 57).

Several limitations of this study warrant consideration. First, convenience sampling from tertiary hospitals limits generalizability; stratified analysis by hospital tier or region was not performed. Recruitment through the China Transplant Pharmacist Alliance may have introduced selection bias. Second, self-reported data may introduce social desirability bias. These scores reflect perceived rather than directly observed practice. No objective clinical performance measures were collected to validate self-reported practices. Third, the cross-sectional design precludes causal inference; associations observed do not imply causality. Fourth, the scoring rule for multiple-choice knowledge items may underestimate true knowledge levels. Fifth, wide confidence intervals for work time and age reflect sparse data. These estimates should be interpreted cautiously. Sixth, the classification thresholds for high, medium, and low score levels lack a universally accepted reference standard. These cut-offs are based on the Chinese medical education grading system rather than a formally validated standard. Seventh, residual confounding may persist from unmeasured variables (e.g., institutional policies, workload, interdisciplinary dynamics, decision-support tools). Eighth, metabolic risks and management priorities may differ by transplant type, and future studies should examine these differences.

## 5 Conclusion

In summary, this first KAP survey using a nationwide convenience sample among Chinese SOT medical staff identifies a gap between positive attitudes and suboptimal knowledge and self-reported practice in managing post-transplant metabolic complications. By revealing role-specific associations of performance, our findings highlight self-reported educational needs and perceived practice gaps that may inform future training or quality-improvement initiatives. Whether these efforts would improve long-term transplant outcomes remains to be determined.

## Data Availability

The original contributions presented in the study are included in the article/Supplementary material, further inquiries can be directed to the corresponding author/s.
