# Supplementary material for: Knowledge, attitude and practice regarding metabolic complication management after solid organ transplantation among medical staff in China
Source: Front Public Health. 2026 Jul 14;14:1784412. doi: 10.3389/fpubh.2026.1784412 (PMC13408235; doi:10.3389/fpubh.2026.1784412)
Supplement: Supplementary file 1 [file Data_Sheet_1.doc]

Supplemental Digital Content

**<Supplementary Methods>**

The detailed questionnaire development and validation

**<Supplementary Tables>**

**Table S1.** “Knowledge-attitude-practice” questionnaire regarding management of metabolic complications after SOT in medical staff

**Table S2.** STROBE Statement—checklist of items that should be included in reports of observational studies

**Table S3.** 5 points N (%) for 12 behavioral items of clinician and clinical pharmacists

<Supplementary Methods>

**The detailed questionnaire development and validation**

1. **Item generation:** An initial 53-item pool was developed based on literature review and research team discussions within the KAP framework (knowledge: 29 items, attitude: 12 items, practice: 12 items).
2. **Delphi method:** Two Delphi rounds were conducted with 15 experts from clinical medicine, clinical pharmacy, and medical education. Expert authority coefficients were 0.94 and 0.95, and the response rate was 100% in both rounds. Kendall‘s W increased from 0.172 (first round) to 0.683 (second round), indicating strong expert consensus. After two rounds of Delphi consultation, the questionnaire consisted of 26 knowledge items, 12 attitude items, and 12 practice items (50 items in total).
3. **Item analysis:** Six items were removed due to poor discriminant validity (*t* value < 3.0, *p* > 0.05), low item-total correlation (*r* < 0.3), or negative impact on Cronbach's α.
4. **Validity:** The overall content validity index (CVI) was 0.939 (item-level CVI range: 0.800–1.000). Exploratory factor analysis (EFA) demonstrated that the data were suitable for factor analysis (KMO = 0.942, Bartlett’s test *p* < 0.001). Although initial extraction suggested a five-factor solution (66.562% variance), a three-factor solution was retained for theoretical consistency with the KAP framework, explaining 61.459% of the cumulative variance. Confirmatory factor analysis (CFA) demonstrated acceptable model fit: χ²/df = 2.106, CFI = 0.916, TLI = 0.909, RMSEA = 0.063, SRMR = 0.044.
5. **Reliability:** Cronbach‘s α was 0.956 for the total questionnaire, 0.901 for the knowledge dimension, 0.977 for the attitude dimension, and 0.955 for the practice dimension. Split-half reliability for the full questionnaire (Spearman-Brown coefficient = 0.976) was also satisfactory.
6. **Final questionnaire**. The validated 43-item questionnaire consists of 19 knowledge items, 12 attitude items, and 12 practice items.

**<Supplementary Tables>**

| Table S1 “Knowledge-attitude-practice” questionnaire regarding management of metabolic complications after SOT in medical staff |
| --- |
| **Item** |
| ***Knowledge*** |
| **K1.** The high incidence of metabolic diseases after transplantation significantly affects the recipient's quality of life and long-term survival.  A Yes  B No  C Not sure |
| **K2.** What are the risk factors associated with metabolic diseases after transplantation?  A. Obesity  B. Donor factors  C. Immunosuppressants  D. Transplant organ function  E. Not sure |
| **K3.** What are the common metabolic complications in transplant recipients?   1. Hypertension 2. Diabetes 3. Dyslipidemia 4. Hyperuricemia 5. Not sure |
| **K4.** Transplant recipients should routinely undergo screening for blood pressure, blood glucose, blood lipids, and uric acid levels.  A. Yes  B. No  C. Not sure |
| **K5.** The target for blood pressure control in transplant recipients should be individualized based on clinical circumstances.  A. Yes  B. No  C. Not sure |
| **K6.** ACEI or ARB medications have the effect of reducing urinary protein.   1. Yes 2. No 3. Not sure |
| **K7.** Which of the following are common adverse reactions of CCB drugs?  A. Headache  B. Edema  C. Facial Flushing  D. Gingival Hyperplasia  E. Not Sure |
| **K8.** Which of the following parameters should be regularly monitored during the use of ACEIs and ARBs?  A. Blood potassium  B. Serum creatinine  C. Blood glucose  D. Not sure |
| **K9.** Regarding the long-term blood glucose control targets for PTDM patients, which of the following is correct?  A. FPG <7.0 mmol/L  B. Postprandial blood glucose <10.0 mmol/L  C. HbAlc <6.0%  D. Targets may be appropriately relaxed for elderly patients or those in poor baseline condition  E. Avoid hypoglycemia  F. Not sure |
| **K10.** In which of the following situations is metformin contraindicated?  A. eGFR <45 mL/(min·1.73 m²)  B. Moderate to severe hepatic impairment  C. Ketoacidosis  D. Severe infection  E. Not sure |
| **K11.** What are the indications for initiating insulin therapy in patients with PTDM?  A. When blood glucose remains uncontrolled on the basis of lifestyle and non-insulin glucose-lowering drugs  B. In the presence of stress conditions, such as severe infection or surgery  C. At the time of PTDM diagnosis, if HbAlc ≥9.0% or FPG ≥11.1 mmol/L, along with obvious hyperglycemic symptoms  D. PTDM complicated by wasting or malnutrition  E. Not sure |
| **K12.** Dyslipidemia in transplant recipients refers to elevated levels of TC and/or TG in the serum, as well as various lipid abnormalities including LDL-C and HDL-C.  A Yes  B No  C Not sure |
| **K13.** The goals and intensity of lipid-lowering therapy for post-transplant patients with dyslipidemia should be determined based on their ASCVD risk stratification.  A Yes  B No  C Not sure |
| **K14.** When pharmacologic therapy is indicated for hypercholesterolemia in transplant recipients, statins are the first-line agents.  A Yes  B No  C Not sure |
| **K15.** Which of the following parameters should be monitored during statin therapy?  A. Blood lipids  B. Liver function  C. Creatine kinase  D. Immunosuppressant blood concentration  E. Not sure |
| **K16.** The intervention threshold for hyperuricemia after transplantation is: serum uric acid >420 µmol/L in men and >360 µmol/L in women.  A Yes  B No  C Not sure |
| **K17.** Regarding the control targets for hyperuricemia after transplantation, which of the following statements are correct?  A For patients with cardiovascular risk factors or cardiovascular disease, SUA should be maintained long-term at <360 μmol/L  B For patients with gout attacks, SUA should be maintained long-term at <300 μmol/L  C Medication should not be used to maintain SUA long-term at <180 μmol/L  D Not sure |
| **K18.** Which of the following are intervention measures for metabolic disorders in transplant recipients?  A. Lifestyle modification  B. Optimizing immunosuppressive regimen based on differences in metabolic adverse effects  C. Initiating pharmacologic therapy when lifestyle changes and immunosuppressant adjustments fail to achieve target levels  D. Not sure |
| **K19.** What do lifestyle modifications for transplant recipients include?  A Reasonable diet  B Appropriate physical activity  C Weight control  D Smoking cessation and alcohol limitation  E Not sure |
| ***Attitude*** |
| **A1.** Timely diagnosis and treatment of metabolic diseases in transplant recipients can prevent and delay the progression of metabolic complications, effectively improving long-term outcomes for recipients.  A Completely disagree  B Disagree  C Uncertain  D Agree  E Completely agree |
| **A2.** Physicians and pharmacists should actively study guidelines or consensus statements on the prevention and management of metabolic disorders in transplant recipients in order to provide better clinical care.  A Completely disagree  B Disagree  C Uncertain  D Agree  E Completely agree |
| **A3.** Establishing standardized procedures for managing metabolic diseases in transplant recipients is very important.  A Completely disagree  B Disagree  C Uncertain  D Agree  E Completely agree |
| **A4.** Evaluating risk factors associated with metabolic diseases in transplant recipients and implementing active interventions can reduce their incidence risk.  A Completely disagree  B Disagree  C Uncertain  D Agree  E Completely agree |
| **A5.** Metabolic-related indicators should be regularly monitored for every transplant recipient.  A Completely disagree  B Disagree  C Uncertain  D Agree  E Completely agree |
| **A6.** Personalized guidance on diet and exercise should be provided to patients.  A Completely disagree  B Disagree  C Uncertain  D Agree  E Completely agree |
| **A7.** The scientific selection and appropriate adjustment of immunosuppressants for transplant recipients are important components of metabolic disease management.  A Completely disagree  B Disagree  C Uncertain  D Agree  E Completely agree |
| **A8.** If lifestyle modifications and adjustments to the immunosuppressive regimen still fail to bring metabolic indicators of transplant recipients to target levels, pharmacological treatment should be initiated promptly.  A Completely disagree  B Disagree  C Uncertain  D Agree  E Completely agree |
| **A9.** Potential interactions between medications for metabolic diseases and immunosuppressants should be thoroughly understood.  A Completely disagree  B Disagree  C Uncertain  D Agree  E Completely agree |
| **A10.** Adverse reactions to medications used for treating metabolic diseases in transplant recipients should be proactively monitored.  A Completely disagree  B Disagree  C Uncertain  D Agree  E Completely agree |
| **A11.** It is very important to regularly provide transplant recipients with education on metabolic diseases.  A Completely disagree  B Disagree  C Uncertain  D Agree  E Completely agree |
| **A12.** In clinical practice, establish open, effective communication with recipients, listen to their needs and concerns, and offer reassurance and hope.  A Completely disagree  B Disagree  C Uncertain  D Agree  E Completely agree |
| ***Practice*** |
| **P1.** Do you proactively learn knowledge about the prevention and treatment of metabolic diseases in transplant recipients?  A Never  B Occasionally  C Sometimes  D Often  E Always |
| **P2.** Do you regularly monitor the blood pressure, blood glucose, lipid levels, and uric acid levels of transplant recipients?  A Never  B Occasionally  C Sometimes  D Often  E Always |
| **P3.** Will you assess the risk of metabolic diseases in transplant recipients and take appropriate measures?  A Never  B Occasionally  C Sometimes  D Often  E Always |
| **P4.** Do you actively intervene in transplant patients with abnormal metabolic indicators?  A Never  B Occasionally  C Sometimes  D Often  E Always |
| **P5.** Will you evaluate the treatment efficacy for metabolic diseases in transplant recipients?  A Never  B Occasionally  C Sometimes  D Often  E Always |
| **P6.** Do you develop individualized control goals for transplant patients with metabolic diseases?  A Never  B Occasionally  C Sometimes  D Often  E Always |
| **P7.** Do you provide dietary and exercise guidance for patients with post-transplant metabolic diseases?  A Never  B Occasionally  C Sometimes  D Often  E Always |
| **P8.** Do you consider adjusting immunosuppressants as a key strategy in managing post-transplant metabolic diseases?  A Never  B Occasionally  C Sometimes  D Often  E Always |
| **P9.** Do you assess the interactions between medications for metabolic diseases and immunosuppressants in transplant recipients?  A Never  B Occasionally  C Sometimes  D Often  E Always |
| **P10.** Do you monitor for adverse reactions to medications used to treat metabolic diseases in transplant recipients?  A Never  B Occasionally  C Sometimes  D Often  E Always |
| **P11.** Do you provide education to transplant recipients about metabolic complications?  A Never  B Occasionally  C Sometimes  D Often  E Always |
| **P12.** During consultations, do you patiently listen to patients’ feelings and concerns and provide them with emotional support?  A Never  B Occasionally  C Sometimes  D Often  E Always |

Table S2. STROBE Statement—checklist of items that should be included in reports of observational studies

|  | **Item No.** | **Recommendation** | **Page  No.** | **Relevant text from manuscript** |
| --- | --- | --- | --- | --- |
| **Title and abstract** | 1 | (*a*) Indicate the study’s design with a commonly used term in the title or the abstract | page 1, 2 | Title: “Knowledge, attitude and practice”; Abstract– Methods: “Utilizing data from a nationwide convenience sample”, “cross‑sectional survey” |
|  |  | (*b*) Provide in the abstract an informative and balanced summary of what was done and what was found | page 2 | Abstract: all sections (Background, Methods, Results, Conclusions) |
| **Introduction** | | | |  |
| Background/rationale | 2 | Explain the scientific background and rationale for the investigation being reported | page 3,4 | Introduction |
| Objectives | 3 | State specific objectives, including any prespecified hypotheses | page 4 | Introduction, last paragraph |
| **Methods** | | | |  |
| Study design | 4 | Present key elements of study design early in the paper | page 4 | Methods 2.1: This study utilized data from a cross‑sectional survey originally designed to develop and validate a KAP questionnaire. |
| Setting | 5 | Describe the setting, locations, and relevant dates, including periods of recruitment, exposure, follow-up, and data collection | page 4,6,7 | Methods 2.1: February to April 2025;  Methods 2.3: online survey, recruitment through China Transplant Pharmacist Alliance.  Result 3.1: 27 provinces across China, six types of solid organ transplantation |
| Participants | 6 | (*a*) *Cohort study*—Give the eligibility criteria, and the sources and methods of selection of participants. Describe methods of follow-up  *Case-control study*—Give the eligibility criteria, and the sources and methods of case ascertainment and control selection. Give the rationale for the choice of cases and controls  *Cross-sectional study*—Give the eligibility criteria, and the sources and methods of selection of participants | Page 5 | Methods 2.2: Convenience sampling, clinicians and clinical pharmacists, from tertiary hospitals; inclusion/exclusion criteria (≥1 year experience, voluntary, no advanced training). |
|  |  | (*b*) *Cohort study*—For matched studies, give matching criteria and number of exposed and unexposed  *Case-control study*—For matched studies, give matching criteria and the number of controls per case |  |  |
| Variables | 7 | Clearly define all outcomes, exposures, predictors, potential confounders, and effect modifiers. Give diagnostic criteria, if applicable | Page 5-7 | Methods 2.2: “gender, age, education, work time, profession, technical title” as independent variables.  Methods 2.4: definition of low/medium/high levels based on percentage thresholds.  Methods 2.5: statistical methods for adjustment. |
| Data sources/ measurement | 8* | For each variable of interest, give sources of data and details of methods of assessment (measurement). Describe comparability of assessment methods if there is more than one group | Page 5 | Methods 2.2: description of knowledge, attitude, practice scoring (Likert scales, formula scoring)  Supplementary file Table S1 provides the full questionnaire. |
| Bias | 9 | Describe any efforts to address potential sources of bias | Page 18 | Discuss-Limitations: self‑report bias, social desirability, selection bias (transplant pharmacist network), convenience sampling, lack of objective measures. |
| Study size | 10 | Explain how the study size was arrived at | Page 5 | Methods 2.2: sample size calculation using Bujang formula (precision 0.07, 95% CI, expected high‑level rate 50%, 20% non‑response). Final n=277. |

| Quantitative variables | 11 | Explain how quantitative variables were handled in the analyses. If applicable, describe which groupings were chosen and why | Page 6 | Methods 2.4: classification of scores into low (<60%), medium (60‑80%), high (≥80%) based on maximum possible score. |
| --- | --- | --- | --- | --- |
| Statistical methods | 12 | (*a*) Describe all statistical methods, including those used to control for confounding | Page 7 | Methods 2.5: Spearman’s correlation, multinomial logistic regression with low and medium as reference categories, VIF collinearity diagnostics, merging of title categories for quasi‑complete separation. |
|  |  | (*b*) Describe any methods used to examine subgroups and interactions | Page 10 | Results 3.5: analyses for each KAP dimension separately; also separate comparisons (medium vs low, high vs low, high vs medium). |
|  |  | (*c*) Explain how missing data were addressed | Page 7 | Results 3.1: only valid questionnaires included; online survey required responses to all questions, so no missing data. |
|  |  | (*d*) *Cohort study*—If applicable, explain how loss to follow-up was addressed  *Case-control study*—If applicable, explain how matching of cases and controls was addressed  *Cross-sectional study*—If applicable, describe analytical methods taking account of sampling strategy |  |  |
|  |  | (*e*) Describe any sensitivity analyses | Page 11 | Results 3.5: “However, this estimate is unstable due to sparse data … should not be interpreted as a reliable negative association.” Also supplementary analysis with “Pass” as reference (Table 10). |
| **Results** | | | | |
| Participants | 13* | (a) Report numbers of individuals at each stage of study—eg numbers potentially eligible, examined for eligibility, confirmed eligible, included in the study, completing follow-up, and analysed | Page 7 | Results 3.1: “A total of 305 questionnaires were distributed. After excluding 28 invalid … 277 valid questionnaires were included … effective response rate 90.82%.” |
|  |  | (b) Give reasons for non-participation at each stage | Page 7 | Results 3.1: “After excluding 28 invalid questionnaires from participants who were not SOT medical staff.” |
|  |  | (c) Consider use of a flow diagram |  |  |
| Descriptive data | 14* | (a) Give characteristics of study participants (eg demographic, clinical, social) and information on exposures and potential confounders | Page 7 | Table 1: demographic characteristics stratified by KAP scores. Also Results 3.1 text. |
|  |  | (b) Indicate number of participants with missing data for each variable of interest | Page 7 | Results 3.1: no missing data reported; online survey required responses. |
|  |  | (c) *Cohort study*—Summarise follow-up time (eg, average and total amount) |  |  |
| Outcome data | 15* | *Cohort study*—Report numbers of outcome events or summary measures over time |  |  |
|  |  | *Case-control study—*Report numbers in each exposure category, or summary measures of exposure |  |  |
|  |  | *Cross-sectional study—*Report numbers of outcome events or summary measures | Page 8.9 | Results 3.2: median KAP scores; Table 2 (in table-clean.doc): low, medium, high rates for each dimension. Also Results 3.3: correct rates, median scores, etc. |
| Main results | 16 | (*a*) Give unadjusted estimates and, if applicable, confounder-adjusted estimates and their precision (eg, 95% confidence interval). Make clear which confounders were adjusted for and why they were included | Page 10,11 | Tables 8‑10: unadjusted and adjusted ORs with 95% CIs. Results 3.5 describes which variables were adjusted. |
|  |  | (*b*) Report category boundaries when continuous variables were categorized | Page 6 | Methods 2.4: low (<60%), medium (60‑80%), high (≥80%). |
|  |  | (*c*) If relevant, consider translating estimates of relative risk into absolute risk for a meaningful time period |  |  |

| Other analyses | 17 | Report other analyses done—eg analyses of subgroups and interactions, and sensitivity analyses | Page 11 | Results 3.5: high vs medium analysis (Table 10); sensitivity for sparse data. |
| --- | --- | --- | --- | --- |
| **Discussion** | | | | |
| Key results | 18 | Summarise key results with reference to study objectives | Page 11 | Discussion first paragraph and throughout. |
| Limitations | 19 | Discuss limitations of the study, taking into account sources of potential bias or imprecision. Discuss both direction and magnitude of any potential bias | Page 18,19 | Discussion – Limitations: convenience sampling, self‑report, cross‑sectional design, scoring rule, sparse data, thresholds, residual confounding, lack of stratification, selection bias. |
| Interpretation | 20 | Give a cautious overall interpretation of results considering objectives, limitations, multiplicity of analyses, results from similar studies, and other relevant evidence | Page 11-19 | Discussion and Conclusion |
| Generalisability | 21 | Discuss the generalisability (external validity) of the study results | Page 18 | Limitations: “convenience sampling from tertiary hospitals limits generalizability”; also mention 27 provinces covered but not fully representative. |
| **Other information** | |  | | |
| Funding | 22 | Give the source of funding and the role of the funders for the present study and, if applicable, for the original study on which the present article is based | Page 20 | Funding |

*Give information separately for cases and controls in case-control studies and, if applicable, for exposed and unexposed groups in cohort and cross-sectional studies.

**Note:** An Explanation and Elaboration article discusses each checklist item and gives methodological background and published examples of transparent reporting. The STROBE checklist is best used in conjunction with this article (freely available on the Web sites of PLoS Medicine at http://www.plosmedicine.org/, Annals of Internal Medicine at http://www.annals.org/, and Epidemiology at http://www.epidem.com/). Information on the STROBE Initiative is available at www.strobe-statement.org.

Table S3. 5 points N (%) for 12 behavioral items of clinician and clinical pharmacists

| Item | Clinician | Clinical pharmacist |
| --- | --- | --- |
| P1 | 19.0% | 17.7% |
| P2 | 45.5% | 29.0% |
| P3 | 35.8% | 27.4% |
| P4 | 39.0% | 25.8% |
| P5 | 39.0% | 30.6% |
| P6 | 35.3% | 25.8% |
| P7 | 36.2% | 27.4% |
| P8 | 33.0% | 17.7% |
| P9 | 38.1% | 29.0% |
| P10 | 39.5% | 24.1% |
| P11 | 33.9% | 22.5% |
| P12 | 37.2% | 30.6% |
